# Diiodido(2,3,5,6-tetrapyridin-2-yl­pyrazine-κ^3^ *N* ^2^,*N* ^1^,*N* ^6^)zinc(II)

**DOI:** 10.1107/S1600536810046842

**Published:** 2010-11-20

**Authors:** Mohammad Yousefi

**Affiliations:** a Islamic Azad University, Shahr-e-Rey Branch, Tehran, Iran

## Abstract

In the title compound, [ZnI_2_(C_24_H_16_N_6_)], the Zn^II^ ion is five-coordinated in a distorted trigonal-bipyramidal geometry by an *N*,*N*,*N*-tridentate 2,3,5,6-tetra-2-pyridinylpyrazine ligand and two iodide ions. The I^−^ ions both occupy equatorial sites. Within the ligand, the dihedral angles between the central pyrazine ring and the two chelating pyridine (py) rings are 14.74 (17) and 26.72 (18)°. The equivalent angles for the non-coordinating py rings are 28.63 (16) and 42.19 (17)°. There is no aromatic π–π stacking in the crystal.

## Related literature

For the synthesis of the ligand, see: Goodwin & Lyons (1959 ▶). For the structure of the free ligand, see: Bock *et al.* (1992 ▶); Greaves & Stoeckli-Evans (1992 ▶). For related structures, see: Ahmadi *et al.* (2010 ▶); Alizadeh *et al.* (2009 ▶); Carranza *et al.* (2004 ▶); Graf *et al.* (1993 ▶, 1997 ▶); Hadadzadeh *et al.* (2006 ▶); Laine *et al.* (1995 ▶); Morsali & Ramazani (2005 ▶); Sakai & Kurashima (2003 ▶); Seyed Sadjadi *et al.* (2008 ▶); Yamada *et al.* (2000 ▶); Zhang *et al.* (2005 ▶).
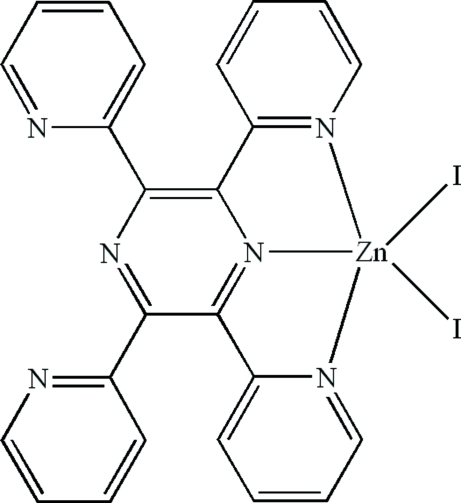


## Experimental

### 

#### Crystal data

[ZnI_2_(C_24_H_16_N_6_)]
*M* *_r_* = 707.60
Triclinic, 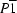

*a* = 10.659 (2) Å
*b* = 10.770 (2) Å
*c* = 12.277 (3) Å
α = 64.31 (3)°
β = 82.41 (3)°
γ = 77.71 (3)°
*V* = 1239.7 (6) Å^3^
*Z* = 2
Mo *K*α radiation
μ = 3.50 mm^−1^
*T* = 120 K
0.49 × 0.35 × 0.30 mm

#### Data collection

Bruker SMART CCD diffractometer
Absorption correction: multi-scan (*SADABS*; Bruker, 1998 ▶) *T*_min_ = 0.240, *T*_max_ = 0.352
14023 measured reflections
6625 independent reflections
6259 reflections with *I* > 2σ(*I*)
*R*_int_ = 0.048

#### Refinement

*R*[*F* ^2^ > 2σ(*F* ^2^)] = 0.037
*wR*(*F* ^2^) = 0.097
*S* = 1.11
6625 reflections
298 parameters
H-atom parameters constrained
Δρ_max_ = 2.47 e Å^−3^
Δρ_min_ = −2.65 e Å^−3^

### 

Data collection: *SMART* (Bruker, 1998 ▶); cell refinement: *SAINT* (Bruker, 1998 ▶); data reduction: *SAINT*; program(s) used to solve structure: *SHELXTL* (Sheldrick, 2008 ▶); program(s) used to refine structure: *SHELXTL*; molecular graphics: *ORTEP-3* (Farrugia, 1997 ▶); software used to prepare material for publication: *WinGX* (Farrugia, 1999 ▶).

## Supplementary Material

Crystal structure: contains datablocks I, global. DOI: 10.1107/S1600536810046842/hb5732sup1.cif

Structure factors: contains datablocks I. DOI: 10.1107/S1600536810046842/hb5732Isup2.hkl

Additional supplementary materials:  crystallographic information; 3D view; checkCIF report

## Figures and Tables

**Table:** 

Zn1—N1	2.207 (3)
Zn1—N3	2.137 (2)
Zn1—N5	2.184 (3)
Zn1—I2	2.5691 (8)
Zn1—I1	2.5888 (10)

**Table:** 

N3—Zn1—N5	74.10 (10)
N3—Zn1—N1	73.73 (10)
N5—Zn1—N1	147.80 (9)
N3—Zn1—I2	125.51 (7)
N5—Zn1—I2	102.61 (8)
N1—Zn1—I2	97.18 (8)
N3—Zn1—I1	119.54 (8)
N5—Zn1—I1	96.46 (8)
N1—Zn1—I1	97.92 (8)
I2—Zn1—I1	114.90 (3)

**Fig. 1.**
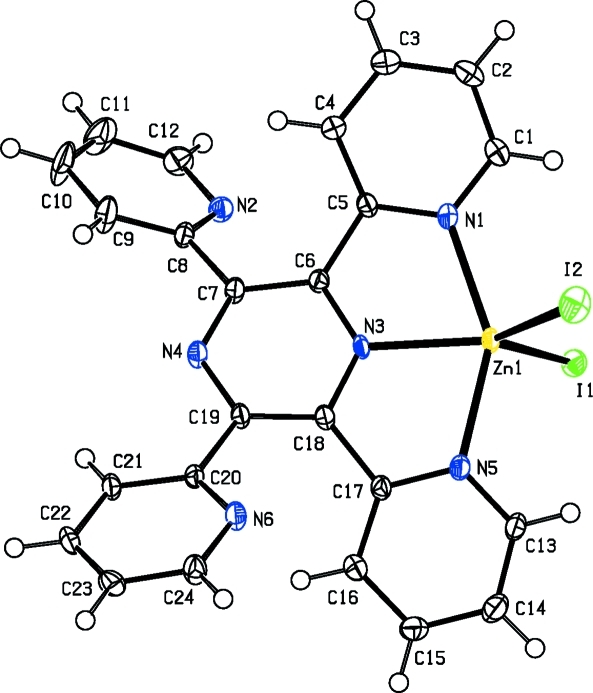
The unit-cell of the title molecule, with the atom-numbering scheme. Displacement ellipsoids are drawn at the 50% probability level.

**Fig. 2.**
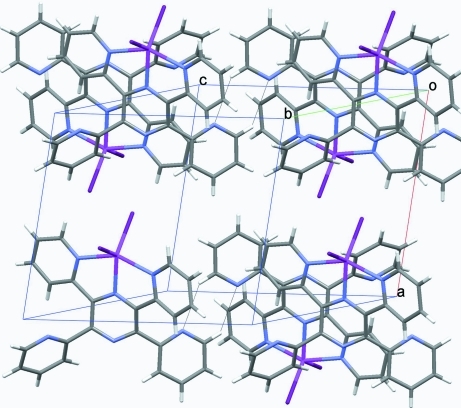
The unit-cell packing diagram for the title molecule.

## supplementary crystallographic information

### Comment

Goodwin & Lyons (1959) were reported the synthesis of 2,3,5,6-tetra-2-pyridinyl-pyrazine (tppz). Bock *et al.* (1992) and Greaves & Stoeckli-Evans (1992) were determined the structure of tppz by single-crystal X-ray diffraction methods. tppz is a good bis-tridentate bridging ligand, and numerous complexes with tppz have been prepared, such as that of ruthenium (Hadadzadeh *et al.*, 2006), platinum (Sakai & Kurashima, 2003), mercury (Zhang *et al.*, 2005), copper (Carranza *et al.*, 2004), iron (Laine *et al.*, 1995), nickel (Graf *et al.*, 1997), palladium (Yadama *et al.*, 2000), cadmium (Seyed Sadjadi *et al.*, 2008) and Lead (Morsali & Ramazani, 2005). For further investigation of 2,3,5,6-tetra-2-pyridinyl-pyrazine, we synthesis the title complex, and report herein in crystal structure.

In the title compound, (Fig. 1), the Zn^II^ atom is five-coordinated in a distorted trigonal-bipyramidal configuration by three N atoms from one 2,3,5,6-tetra-2-pyridinyl-pyrazine and two terminal I. The Zn—N and Zn—I bond lengths and angles (Table 1) are within normal range of [ZnCl_2_(tppz)], (Graf *et al.*, 1993), [ZnBr_2_(tppz)], (Ahmadi *et al.*, 2010) and [ZnI_2_(6,6'-dmbpy)], (Alizadeh *et al.*, 2009) [where 6,6'-dmbpy is 6,6'-dimethyl-2, 2'-bipyridine] respectively.

### Experimental

For the preparation of the title compound, a solution of 2,3,5,6-tetra-2-pyridinyl-pyrazine (0.60 g, 1.5 mmol) in HCCl_3_ (25 ml) was added to a solution of ZnI_2_ (0.48 g, 1.50 mmol) in methanol (25 ml) at room temperature. The suitable crystals for X-ray diffraction experiment were obtained by methanol diffusion to a colorless solution in DMSO. Yellow blocks were isolated after one week (yield; 0.81 g, 76.3%).

### Refinement

All H atoms were positioned geometrically, with C—H=0.93Å for aromatics H and constrained to ride on their parent atoms, with *U*_iso_(H)=1.2*U*_eq_.

### Crystal data

**Table:**

[ZnI_2_(C_24_H_16_N_6_)]	*Z* = 2
*M**_r_* = 707.60	*F*(000) = 676
Triclinic, *P*1	*D*_x_ = 1.895 Mg m^−^^3^
Hall symbol: -P 1	Mo *K*α radiation, λ = 0.71073 Å
*a* = 10.659 (2) Å	Cell parameters from 14023 reflections
*b* = 10.770 (2) Å	θ = 2.2–29.2°
*c* = 12.277 (3) Å	µ = 3.50 mm^−^^1^
α = 64.31 (3)°	*T* = 120 K
β = 82.41 (3)°	Block, yellow
γ = 77.71 (3)°	0.49 × 0.35 × 0.30 mm
*V* = 1239.7 (6) Å^3^	

### Data collection

**Table:**

Bruker SMART CCD diffractometer	6625 independent reflections
Radiation source: fine-focus sealed tube	6259 reflections with *I* > 2σ(*I*)
graphite	*R*_int_ = 0.048
phi and ω scans	θ_max_ = 29.2°, θ_min_ = 2.2°
Absorption correction: multi-scan (*SADABS*; Bruker, 1998)	*h* = −14→14
*T*_min_ = 0.240, *T*_max_ = 0.352	*k* = −14→13
14023 measured reflections	*l* = −16→16

### Refinement

**Table:**

Refinement on *F*^2^	Primary atom site location: structure-invariant direct methods
Least-squares matrix: full	Secondary atom site location: difference Fourier map
*R*[*F*^2^ > 2σ(*F*^2^)] = 0.037	Hydrogen site location: inferred from neighbouring sites
*wR*(*F*^2^) = 0.097	H-atom parameters constrained
*S* = 1.11	*w* = 1/[σ^2^(*F*_o_^2^) + (0.0526*P*)^2^ + 2.2743*P*] where *P* = (*F*_o_^2^ + 2*F*_c_^2^)/3
6625 reflections	(Δ/σ)_max_ = 0.018
298 parameters	Δρ_max_ = 2.47 e Å^−^^3^
0 restraints	Δρ_min_ = −2.65 e Å^−^^3^

### Special details

**Table:**

Geometry. All e.s.d.'s (except the e.s.d. in the dihedral angle between two l.s. planes) are estimated using the full covariance matrix. The cell e.s.d.'s are taken into account individually in the estimation of e.s.d.'s in distances, angles and torsion angles; correlations between e.s.d.'s in cell parameters are only used when they are defined by crystal symmetry. An approximate (isotropic) treatment of cell e.s.d.'s is used for estimating e.s.d.'s involving l.s. planes.
Refinement. Refinement of *F*^2^ against ALL reflections. The weighted *R*-factor *wR* and goodness of fit *S* are based on *F*^2^, conventional *R*-factors *R* are based on *F*, with *F* set to zero for negative *F*^2^. The threshold expression of *F*^2^ > σ(*F*^2^) is used only for calculating *R*-factors(gt) *etc*. and is not relevant to the choice of reflections for refinement. *R*-factors based on *F*^2^ are statistically about twice as large as those based on *F*, and *R*- factors based on ALL data will be even larger.

### Fractional atomic coordinates and isotropic or equivalent isotropic displacement parameters (Å^2^)

**Table:**

	*x*	*y*	*z*	*U*_iso_*/*U*_eq_	
C1	0.3744 (3)	0.1483 (4)	0.0215 (3)	0.0246 (6)	
H1	0.4559	0.1099	0.0521	0.029*	
C2	0.3592 (3)	0.1816 (4)	−0.0992 (3)	0.0256 (6)	
H2	0.4278	0.1627	−0.1481	0.031*	
C3	0.2382 (3)	0.2439 (3)	−0.1441 (3)	0.0207 (6)	
H3	0.2249	0.2688	−0.2249	0.025*	
C4	0.1365 (3)	0.2695 (3)	−0.0691 (3)	0.0182 (5)	
H4	0.0556	0.3145	−0.0991	0.022*	
C5	0.1587 (3)	0.2263 (3)	0.0522 (3)	0.0147 (5)	
C6	0.0586 (3)	0.2431 (3)	0.1435 (3)	0.0141 (5)	
C7	−0.0751 (3)	0.2860 (3)	0.1297 (3)	0.0154 (5)	
C8	−0.1455 (3)	0.2681 (3)	0.0417 (3)	0.0168 (5)	
C9	−0.2477 (3)	0.3674 (4)	−0.0189 (4)	0.0291 (7)	
H9	−0.2744	0.4490	−0.0073	0.035*	
C10	−0.3089 (4)	0.3413 (5)	−0.0977 (4)	0.0403 (10)	
H10	−0.3775	0.4058	−0.1408	0.048*	
C11	−0.2661 (4)	0.2169 (5)	−0.1112 (4)	0.0355 (9)	
H11	−0.3056	0.1970	−0.1635	0.043*	
C12	−0.1640 (3)	0.1237 (4)	−0.0453 (3)	0.0231 (6)	
H12	−0.1363	0.0403	−0.0539	0.028*	
C13	0.2711 (3)	0.0708 (4)	0.5572 (3)	0.0222 (6)	
H13	0.3598	0.0421	0.5554	0.027*	
C14	0.2053 (3)	0.0432 (4)	0.6687 (3)	0.0243 (6)	
H14	0.2493	−0.0019	0.7400	0.029*	
C15	0.0730 (3)	0.0842 (4)	0.6715 (3)	0.0231 (6)	
H15	0.0267	0.0650	0.7450	0.028*	
C16	0.0100 (3)	0.1546 (3)	0.5628 (3)	0.0193 (5)	
H16	−0.0786	0.1835	0.5624	0.023*	
C17	0.0834 (3)	0.1805 (3)	0.4550 (3)	0.0152 (5)	
C18	0.0266 (3)	0.2466 (3)	0.3346 (3)	0.0143 (5)	
C19	−0.0955 (3)	0.3294 (3)	0.3007 (3)	0.0150 (5)	
C20	−0.1726 (3)	0.4133 (3)	0.3629 (3)	0.0146 (5)	
C21	−0.3050 (3)	0.4520 (3)	0.3513 (3)	0.0161 (5)	
H21	−0.3472	0.4211	0.3085	0.019*	
C22	−0.3724 (3)	0.5377 (3)	0.4050 (3)	0.0180 (5)	
H22	−0.4607	0.5672	0.3972	0.022*	
C23	−0.3068 (3)	0.5785 (3)	0.4703 (3)	0.0200 (6)	
H23	−0.3504	0.6345	0.5084	0.024*	
C24	−0.1741 (3)	0.5343 (3)	0.4782 (3)	0.0216 (6)	
H24	−0.1305	0.5611	0.5230	0.026*	
N1	0.2767 (2)	0.1693 (3)	0.0955 (2)	0.0184 (5)	
N2	−0.1032 (2)	0.1480 (3)	0.0301 (2)	0.0172 (5)	
N3	0.1029 (2)	0.2183 (3)	0.2492 (2)	0.0140 (4)	
N4	−0.1457 (2)	0.3385 (3)	0.2030 (2)	0.0158 (4)	
N5	0.2114 (2)	0.1371 (3)	0.4525 (2)	0.0172 (5)	
N6	−0.1068 (2)	0.4546 (3)	0.4237 (3)	0.0187 (5)	
Zn1	0.30085 (3)	0.13103 (3)	0.28390 (3)	0.01425 (8)	
I1	0.373663 (19)	−0.137621 (19)	0.369751 (17)	0.01892 (7)	
I2	0.48284 (2)	0.27145 (2)	0.23922 (2)	0.02865 (7)	

### Atomic displacement parameters (Å^2^)

**Table:**

	*U*^11^	*U*^22^	*U*^33^	*U*^12^	*U*^13^	*U*^23^
C1	0.0166 (14)	0.0320 (17)	0.0233 (15)	0.0029 (12)	0.0006 (11)	−0.0136 (13)
C2	0.0217 (15)	0.0316 (17)	0.0224 (15)	−0.0029 (13)	0.0067 (12)	−0.0133 (13)
C3	0.0238 (15)	0.0206 (13)	0.0167 (13)	−0.0053 (11)	0.0002 (11)	−0.0065 (11)
C4	0.0173 (13)	0.0208 (13)	0.0147 (12)	−0.0013 (10)	−0.0012 (10)	−0.0066 (10)
C5	0.0115 (11)	0.0157 (12)	0.0170 (12)	−0.0004 (9)	−0.0001 (9)	−0.0079 (10)
C6	0.0125 (12)	0.0164 (12)	0.0153 (12)	0.0006 (9)	−0.0029 (9)	−0.0092 (10)
C7	0.0121 (12)	0.0170 (12)	0.0189 (13)	0.0017 (9)	−0.0045 (10)	−0.0101 (10)
C8	0.0129 (12)	0.0209 (13)	0.0177 (13)	0.0024 (10)	−0.0052 (10)	−0.0103 (11)
C9	0.0224 (15)	0.0323 (17)	0.0384 (19)	0.0111 (13)	−0.0165 (14)	−0.0231 (15)
C10	0.0318 (19)	0.048 (2)	0.051 (2)	0.0170 (17)	−0.0308 (19)	−0.033 (2)
C11	0.038 (2)	0.044 (2)	0.037 (2)	0.0005 (17)	−0.0191 (17)	−0.0266 (18)
C12	0.0244 (15)	0.0249 (15)	0.0244 (15)	−0.0054 (12)	−0.0013 (12)	−0.0141 (12)
C13	0.0166 (13)	0.0302 (16)	0.0211 (14)	0.0069 (11)	−0.0084 (11)	−0.0148 (12)
C14	0.0257 (16)	0.0253 (15)	0.0206 (14)	0.0048 (12)	−0.0095 (12)	−0.0101 (12)
C15	0.0252 (15)	0.0259 (15)	0.0160 (13)	0.0011 (12)	−0.0007 (11)	−0.0092 (12)
C16	0.0150 (12)	0.0229 (14)	0.0200 (14)	−0.0003 (11)	−0.0001 (10)	−0.0106 (11)
C17	0.0128 (12)	0.0173 (12)	0.0177 (13)	0.0024 (9)	−0.0031 (10)	−0.0107 (10)
C18	0.0104 (11)	0.0168 (12)	0.0183 (13)	−0.0003 (9)	−0.0016 (9)	−0.0104 (10)
C19	0.0101 (11)	0.0186 (12)	0.0190 (13)	0.0018 (9)	−0.0027 (9)	−0.0119 (10)
C20	0.0116 (11)	0.0167 (12)	0.0155 (12)	0.0025 (9)	−0.0028 (9)	−0.0082 (10)
C21	0.0095 (11)	0.0190 (12)	0.0196 (13)	0.0000 (10)	−0.0021 (9)	−0.0088 (11)
C22	0.0103 (11)	0.0191 (13)	0.0224 (13)	−0.0003 (10)	0.0021 (10)	−0.0085 (11)
C23	0.0206 (14)	0.0201 (13)	0.0210 (14)	0.0005 (11)	0.0016 (11)	−0.0127 (11)
C24	0.0174 (14)	0.0262 (15)	0.0272 (15)	0.0033 (11)	−0.0054 (11)	−0.0186 (13)
N1	0.0124 (11)	0.0227 (12)	0.0179 (11)	0.0028 (9)	−0.0029 (9)	−0.0087 (10)
N2	0.0171 (11)	0.0189 (11)	0.0179 (11)	−0.0017 (9)	−0.0026 (9)	−0.0100 (9)
N3	0.0077 (9)	0.0180 (11)	0.0169 (11)	0.0026 (8)	−0.0019 (8)	−0.0098 (9)
N4	0.0119 (10)	0.0169 (11)	0.0208 (12)	0.0015 (8)	−0.0042 (9)	−0.0108 (9)
N5	0.0136 (11)	0.0214 (11)	0.0179 (11)	0.0033 (9)	−0.0045 (9)	−0.0113 (10)
N6	0.0127 (11)	0.0233 (12)	0.0245 (13)	0.0018 (9)	−0.0044 (9)	−0.0154 (10)
Zn1	0.00936 (14)	0.01684 (15)	0.01719 (16)	0.00192 (11)	−0.00304 (11)	−0.00892 (12)
I1	0.02016 (11)	0.01584 (10)	0.02007 (10)	0.00168 (7)	−0.00295 (7)	−0.00848 (7)
I2	0.02559 (12)	0.02774 (12)	0.03527 (13)	−0.01186 (9)	−0.00476 (9)	−0.01144 (10)

### Geometric parameters (Å, °)

**Table:**

C1—N1	1.337 (4)	C14—C15	1.385 (5)
C1—C2	1.389 (5)	C14—H14	0.9300
C1—H1	0.9300	C15—C16	1.396 (4)
C2—C3	1.384 (5)	C15—H15	0.9300
C2—H2	0.9300	C16—C17	1.393 (4)
C3—C4	1.389 (4)	C16—H16	0.9300
C3—H3	0.9300	C17—N5	1.346 (4)
C4—C5	1.391 (4)	C17—C18	1.483 (4)
C4—H4	0.9300	C18—N3	1.341 (4)
C5—N1	1.344 (4)	C18—C19	1.411 (4)
C5—C6	1.485 (4)	C19—N4	1.334 (4)
C6—N3	1.336 (4)	C19—C20	1.481 (4)
C6—C7	1.410 (4)	C20—N6	1.345 (4)
C7—N4	1.332 (4)	C20—C21	1.391 (4)
C7—C8	1.492 (4)	C21—C22	1.387 (4)
C8—N2	1.339 (4)	C21—H21	0.9300
C8—C9	1.383 (4)	C22—C23	1.382 (4)
C9—C10	1.386 (5)	C22—H22	0.9300
C9—H9	0.9300	C23—C24	1.395 (4)
C10—C11	1.394 (6)	C23—H23	0.9300
C10—H10	0.9300	C24—N6	1.344 (4)
C11—C12	1.382 (5)	C24—H24	0.9300
C11—H11	0.9300	Zn1—N1	2.207 (3)
C12—N2	1.337 (4)	Zn1—N3	2.137 (2)
C12—H12	0.9300	Zn1—N5	2.184 (3)
C13—N5	1.339 (4)	Zn1—I2	2.5691 (8)
C13—C14	1.392 (5)	Zn1—I1	2.5888 (10)
C13—H13	0.9300		
			
N1—C1—C2	122.7 (3)	C15—C16—H16	120.8
N1—C1—H1	118.7	N5—C17—C16	122.3 (3)
C2—C1—H1	118.7	N5—C17—C18	114.3 (3)
C3—C2—C1	117.7 (3)	C16—C17—C18	123.2 (3)
C3—C2—H2	121.1	N3—C18—C19	117.3 (3)
C1—C2—H2	121.1	N3—C18—C17	113.7 (2)
C2—C3—C4	120.2 (3)	C19—C18—C17	128.9 (3)
C2—C3—H3	119.9	N4—C19—C18	118.9 (3)
C4—C3—H3	119.9	N4—C19—C20	116.1 (2)
C3—C4—C5	118.3 (3)	C18—C19—C20	124.9 (3)
C3—C4—H4	120.8	N6—C20—C21	123.3 (3)
C5—C4—H4	120.8	N6—C20—C19	116.3 (2)
N1—C5—C4	121.6 (3)	C21—C20—C19	120.3 (3)
N1—C5—C6	114.1 (3)	C22—C21—C20	118.4 (3)
C4—C5—C6	124.3 (3)	C22—C21—H21	120.8
N3—C6—C7	117.3 (3)	C20—C21—H21	120.8
N3—C6—C5	115.1 (2)	C23—C22—C21	119.1 (3)
C7—C6—C5	127.6 (3)	C23—C22—H22	120.4
N4—C7—C6	119.4 (3)	C21—C22—H22	120.4
N4—C7—C8	116.8 (2)	C22—C23—C24	118.9 (3)
C6—C7—C8	123.7 (3)	C22—C23—H23	120.6
N2—C8—C9	124.2 (3)	C24—C23—H23	120.6
N2—C8—C7	114.2 (2)	N6—C24—C23	122.7 (3)
C9—C8—C7	121.6 (3)	N6—C24—H24	118.6
C8—C9—C10	117.7 (3)	C23—C24—H24	118.6
C8—C9—H9	121.1	C1—N1—C5	119.3 (3)
C10—C9—H9	121.1	C1—N1—Zn1	123.3 (2)
C9—C10—C11	119.0 (3)	C5—N1—Zn1	117.2 (2)
C9—C10—H10	120.5	C12—N2—C8	117.4 (3)
C11—C10—H10	120.5	C6—N3—C18	122.0 (2)
C12—C11—C10	118.8 (3)	C6—N3—Zn1	119.27 (19)
C12—C11—H11	120.6	C18—N3—Zn1	118.77 (19)
C10—C11—H11	120.6	C7—N4—C19	120.4 (2)
N2—C12—C11	122.9 (3)	C13—N5—C17	118.9 (3)
N2—C12—H12	118.5	C13—N5—Zn1	122.5 (2)
C11—C12—H12	118.5	C17—N5—Zn1	116.77 (19)
N5—C13—C14	122.4 (3)	C24—N6—C20	117.5 (3)
N5—C13—H13	118.8	N3—Zn1—N5	74.10 (10)
C14—C13—H13	118.8	N3—Zn1—N1	73.73 (10)
C15—C14—C13	118.8 (3)	N5—Zn1—N1	147.80 (9)
C15—C14—H14	120.6	N3—Zn1—I2	125.51 (7)
C13—C14—H14	120.6	N5—Zn1—I2	102.61 (8)
C14—C15—C16	119.3 (3)	N1—Zn1—I2	97.18 (8)
C14—C15—H15	120.4	N3—Zn1—I1	119.54 (8)
C16—C15—H15	120.4	N5—Zn1—I1	96.46 (8)
C17—C16—C15	118.3 (3)	N1—Zn1—I1	97.92 (8)
C17—C16—H16	120.8	I2—Zn1—I1	114.90 (3)
			
N1—C1—C2—C3	2.6 (5)	C6—C5—N1—Zn1	−5.3 (3)
C1—C2—C3—C4	−0.9 (5)	C11—C12—N2—C8	0.6 (5)
C2—C3—C4—C5	−2.4 (5)	C9—C8—N2—C12	0.2 (5)
C3—C4—C5—N1	4.3 (5)	C7—C8—N2—C12	178.5 (3)
C3—C4—C5—C6	−178.3 (3)	C7—C6—N3—C18	−6.9 (4)
N1—C5—C6—N3	9.1 (4)	C5—C6—N3—C18	171.2 (3)
C4—C5—C6—N3	−168.5 (3)	C7—C6—N3—Zn1	173.1 (2)
N1—C5—C6—C7	−173.1 (3)	C5—C6—N3—Zn1	−8.8 (3)
C4—C5—C6—C7	9.3 (5)	C19—C18—N3—C6	−12.5 (4)
N3—C6—C7—N4	19.3 (4)	C17—C18—N3—C6	165.6 (3)
C5—C6—C7—N4	−158.5 (3)	C19—C18—N3—Zn1	167.5 (2)
N3—C6—C7—C8	−156.8 (3)	C17—C18—N3—Zn1	−14.4 (3)
C5—C6—C7—C8	25.5 (5)	C6—C7—N4—C19	−11.1 (4)
N4—C7—C8—N2	−138.0 (3)	C8—C7—N4—C19	165.1 (3)
C6—C7—C8—N2	38.1 (4)	C18—C19—N4—C7	−8.9 (4)
N4—C7—C8—C9	40.4 (5)	C20—C19—N4—C7	168.9 (3)
C6—C7—C8—C9	−143.5 (3)	C14—C13—N5—C17	1.0 (5)
N2—C8—C9—C10	−0.8 (6)	C14—C13—N5—Zn1	−162.8 (3)
C7—C8—C9—C10	−179.0 (4)	C16—C17—N5—C13	−2.4 (5)
C8—C9—C10—C11	0.6 (7)	C18—C17—N5—C13	−177.3 (3)
C9—C10—C11—C12	0.1 (7)	C16—C17—N5—Zn1	162.4 (2)
C10—C11—C12—N2	−0.7 (7)	C18—C17—N5—Zn1	−12.6 (3)
N5—C13—C14—C15	0.9 (5)	C23—C24—N6—C20	−2.2 (5)
C13—C14—C15—C16	−1.5 (5)	C21—C20—N6—C24	1.8 (5)
C14—C15—C16—C17	0.2 (5)	C19—C20—N6—C24	178.2 (3)
C15—C16—C17—N5	1.7 (5)	C6—N3—Zn1—N5	−173.8 (2)
C15—C16—C17—C18	176.2 (3)	C18—N3—Zn1—N5	6.2 (2)
N5—C17—C18—N3	17.4 (4)	C6—N3—Zn1—N1	4.6 (2)
C16—C17—C18—N3	−157.4 (3)	C18—N3—Zn1—N1	−175.4 (2)
N5—C17—C18—C19	−164.7 (3)	C6—N3—Zn1—I2	91.9 (2)
C16—C17—C18—C19	20.4 (5)	C18—N3—Zn1—I2	−88.2 (2)
N3—C18—C19—N4	20.8 (4)	C6—N3—Zn1—I1	−85.4 (2)
C17—C18—C19—N4	−156.9 (3)	C18—N3—Zn1—I1	94.6 (2)
N3—C18—C19—C20	−156.7 (3)	C13—N5—Zn1—N3	168.2 (3)
C17—C18—C19—C20	25.5 (5)	C17—N5—Zn1—N3	4.1 (2)
N4—C19—C20—N6	−152.2 (3)	C13—N5—Zn1—N1	165.4 (2)
C18—C19—C20—N6	25.4 (4)	C17—N5—Zn1—N1	1.3 (3)
N4—C19—C20—C21	24.3 (4)	C13—N5—Zn1—I2	−68.1 (3)
C18—C19—C20—C21	−158.1 (3)	C17—N5—Zn1—I2	127.8 (2)
N6—C20—C21—C22	0.1 (5)	C13—N5—Zn1—I1	49.3 (3)
C19—C20—C21—C22	−176.1 (3)	C17—N5—Zn1—I1	−114.8 (2)
C20—C21—C22—C23	−1.6 (4)	C1—N1—Zn1—N3	175.7 (3)
C21—C22—C23—C24	1.2 (5)	C5—N1—Zn1—N3	0.7 (2)
C22—C23—C24—N6	0.8 (5)	C1—N1—Zn1—N5	178.5 (2)
C2—C1—N1—C5	−0.8 (5)	C5—N1—Zn1—N5	3.6 (3)
C2—C1—N1—Zn1	−175.6 (3)	C1—N1—Zn1—I2	50.7 (3)
C4—C5—N1—C1	−2.7 (5)	C5—N1—Zn1—I2	−124.2 (2)
C6—C5—N1—C1	179.6 (3)	C1—N1—Zn1—I1	−65.8 (3)
C4—C5—N1—Zn1	172.4 (2)	C5—N1—Zn1—I1	119.3 (2)
